# The RIPK family: expression profile and prognostic value in lung adenocarcinoma

**DOI:** 10.18632/aging.204195

**Published:** 2022-07-30

**Authors:** Guo Li, Zhijie Xu, Jinwu Peng, Yuanliang Yan, Yong Liu, Xin Zhang, Yuanzheng Qiu, Chencheng Fu

**Affiliations:** 1 Department of Otolaryngology Head and Neck Surgery, Xiangya Hospital, Central South University, Changsha 410008, China; 2 Otolaryngology Major Disease Research Key Laboratory of Hunan Province, Xiangya Hospital, Central South University, Changsha 410008, China; 3 Clinical Research Center for Laryngopharyngeal and Voice Disorders in Hunan Province, Xiangya Hospital, Central South University, Changsha 410008, China; 4 Department of Pathology, Xiangya Hospital, Central South University, Changsha 410008, China; 5 Department of Pathology, Xiangya Changde Hospital, Changde 415000, China; 6 National Clinical Research Center for Geriatric Disorders, Xiangya Hospital, Central South University, Changsha 410008, China; 7 Department of Pharmacy, Xiangya Hospital, Central South University, Changsha 410008, China

**Keywords:** lung adenocarcinoma, RIPKs family, expression profiles, immune infiltrating, methylation

## Abstract

Receptor interacting protein kinases (RIPKs) are a family of serine/threonine kinases which are supposed to regulate tumor generation and progression. Rare study illustrates the roles and functions of RIPKs family in lung adenocarcinoma (LUAD) comprehensively. Our results indicated that the expression of RIPK2 higher in LUAD patients while RIPK5 (encoded by gene DSTYK) expression was lower. Only RIPK2 had a strong correlation with pathological stage in LUAD patients. Kaplan-Meier plotter revealed that LUAD patients with low RIPK2 or RIPK3 level showed better overall survival (OS), but worse when LUAD patients with high RIPK5. Further, lower expression of RIPK2 and higher expression of RIPK1, RIPK4 and RIPK5 prompted a longer disease free survival (DFS). Genetic alterations based on cBioPortal revealing 16% alteration rates of RIPK2, as well as RIPK5. We also found that the functions of RIPKs family were linked to cellular senescence, protein serine/threonine kinase activity, apoptosis process et al. TIMER database indicated that the RIPKs family members had distinct relationships with the infiltration of six types of immune cells (macrophages, neutrophils, CD8+ T-cells, B-cells, CD4+ T-cells and dendritic cells). Moreover, RIPK2 could be observed as an independent prognostic factor with Cox proportional hazard model analysis. DiseaseMeth databases revealed that the global methylation levels of RIPK2 increased in LUAD patients. Thus, the findings above will enhance the understanding of RIPKs family in LUAD pathology and progression, providing novel insights into RIPKs-core therapy for LUAD patients.

## INTRODUCTION

Receptor-interacting protein kinases (RIPKs) are known as a family of serine/threonine kinases, including RIPK1, RIPK2, RIPK3, RIPK4 and RIPK5/DSTYK [1]. Their functions, regulation, and pathophysiological roles have largely remained a labyrinth. The proteins of RIPKs family have garnered significant interest because of their role in regulating various forms of cell death, inflammation, and innate immunity [2, 3]. Owing to the exploration of cell death mechanisms in recent years, in addition to classical apoptosis and necroptosis via activation of NF-κB signaling or ERK/JNK signaling [4], new forms of cell death, such as ferroptosis, have been shown to be related to the RIPK family [5]. Furthermore, an increasing number of research teams have focused on the effect of RIPK genes on tumor progression and tumor immunity via regulation of cell death and correlated pathways [3, 6].

Lung carcinoma is reported as an aggressive malignancy in humans, with a high rate of mortality worldwide [7]. Approximately 40% of all non-small cell lung carcinomas (NSCLCs) were finally diagnosed as lung adenocarcinoma (LUAD) [8]. Surgery remains the main treatment option for LUAD, accompanied by various modalities of chemotherapy, irradiation therapy, targeted therapy, and even immunotherapy in recent decades [9, 10]. Despite extensive research regarding the underlying mechanism of development and treatment resistance among patients with LUAD, the 5-year survival rate remains unsatisfactory, and most patients still experience relapse, metastasis, and death [11].

In this study, we examined the roles of the RIPK family in LUAD based on specific online databases (Table 1) that estimate the biological significance and potential functions of RIPK proteins in LUAD.

**Table 1:** The main bioinformatics tools used to analyze the functions of RIPK2 in LUAD.

**Database**	**Samples**	**URL**
GEPIA	Tissues	http://gepia.cancer-pku.cn/
Wanderer	Tissues	http://maplab.imppc.org/wanderer/
UCLCAN	Tissues	http://ualcan.path.uab.edu/index.html
PrognoScan	Tissues	http://dna00.bio.kyutech.ac.jp/PrognoScan/index.html
cBioPortal	Tissues	https://www.cbioportal.org/
STRING	−	https://www.string-db.org/
Cytoscape	−	https://www.cytoscape.org/
WebGestalt		http://www.webgestalt.org/
TIMER	Tissues	https://cistrome.shinyapps.io/timer
DiseaseMeth	Tissues	http://bio-bigdata.hrbmu.edu.cn/diseasemeth/

## RESULTS

### The expression of the RIPK family in LUAD patients

**Figure 1:**
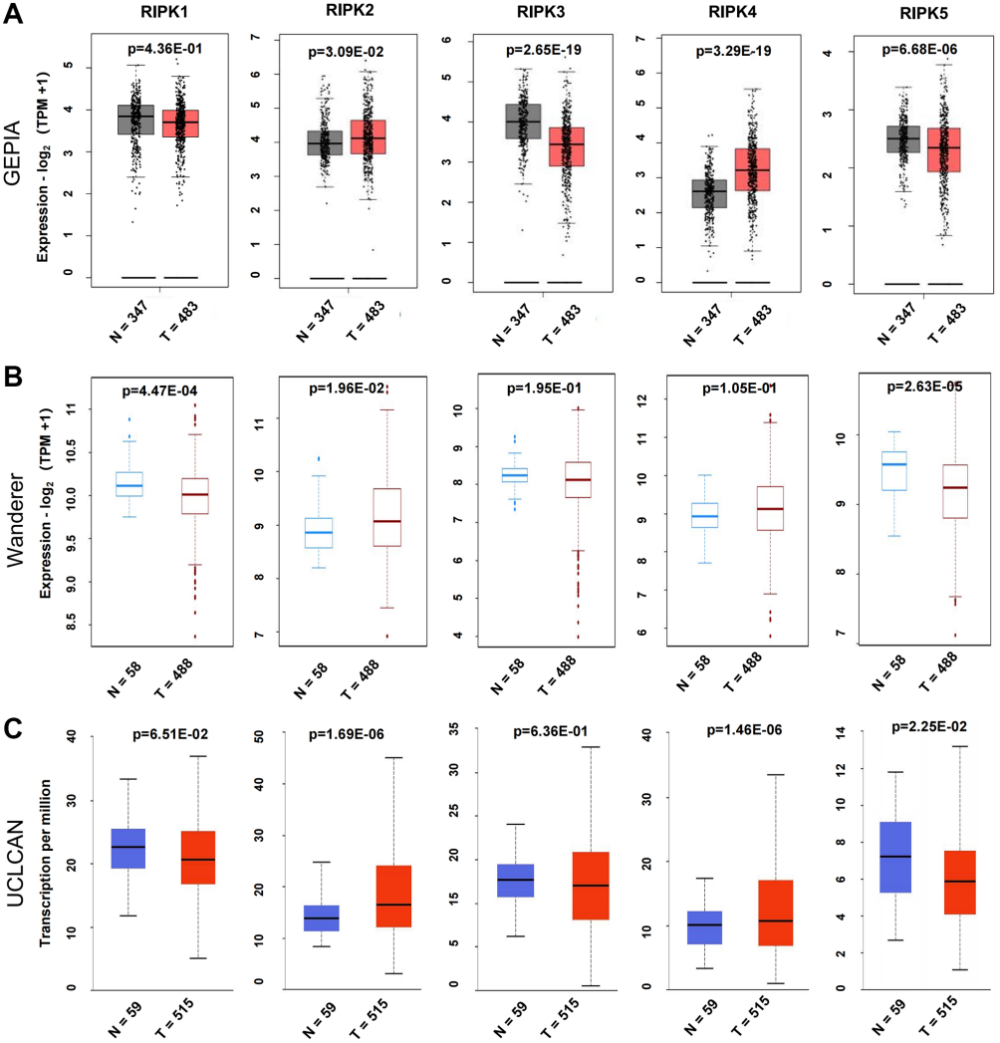
**Differential mRNA expression analysis of the RIPK family in LUAD and normal tissues.** (**A**) The expression profiles were collected from the GEPIA databases. (**B**) The expression profiles were obtained from the Wanderer databases. (**C**) The expression profiles were analysis via the UALCAN databases.

To better understand the RIPK family functions in LUAD patients, we firstly evaluated the mRNA levels of RIPK1, RIPK2, RIPK3, RIPK4 and RIPK5 from databases GEPIA, Wanderer and UCLCAN. When compared to normal tissues, RIPK2 presented coherently upregulated in LUAD tissues among three databases, while RIPK5 (DSTYK) was decreased. Lower expression of RIPK1 were spotted in LUAD tissue from Wanderer and UCLCAN data. GEPIA database illustrated a downregulated RIPK3 in tumor tissue, but no significant change in Wanderer and UCLCAN database. The level of RIPK4 was found increased in LUAD samples base on GEPIA and UCLCAN data, but this trend was not found in Wanderer data (Figure 1A–1C).

**Figure 2:**
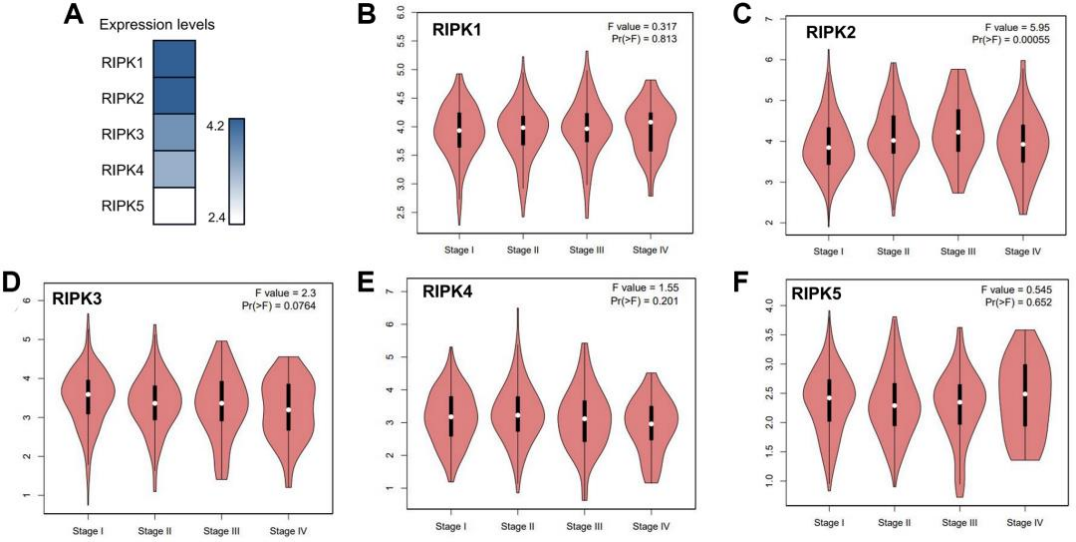
**The relative expression levels of the RIPK family in LUAD patients and their correlation to clinic stages.** (**A**) GEPIA databases were used to evaluate the relative expression levels of the RIPK family in LUAD patients. (**B**–**F**) the correlation between expression of RIPK1-5 and tumor clinic stage.

When evaluating the mRNA levels of the RIPK family in LUAD tissues, RIPK1 and RIPK2 turn out to be the top two expression gene, while RIPK5 expressed lowest (Figure 2A). Further, based on different pathological stages, the mRNAs levels of each RIPK members also analyzed respectively. Only RIPK2 expression positively correlated with pathological stage (*p* = 0.00055) (Figure 2C). No significant correlation observed in other RIPK family and pathological stages (*p* > 0.05, Figure 2B, 2D–2F). The data above implied that RIPK genes could participate in LUAD progression.

### The prognostic value of the RIPK family in LUAD patients

**Figure 3:**
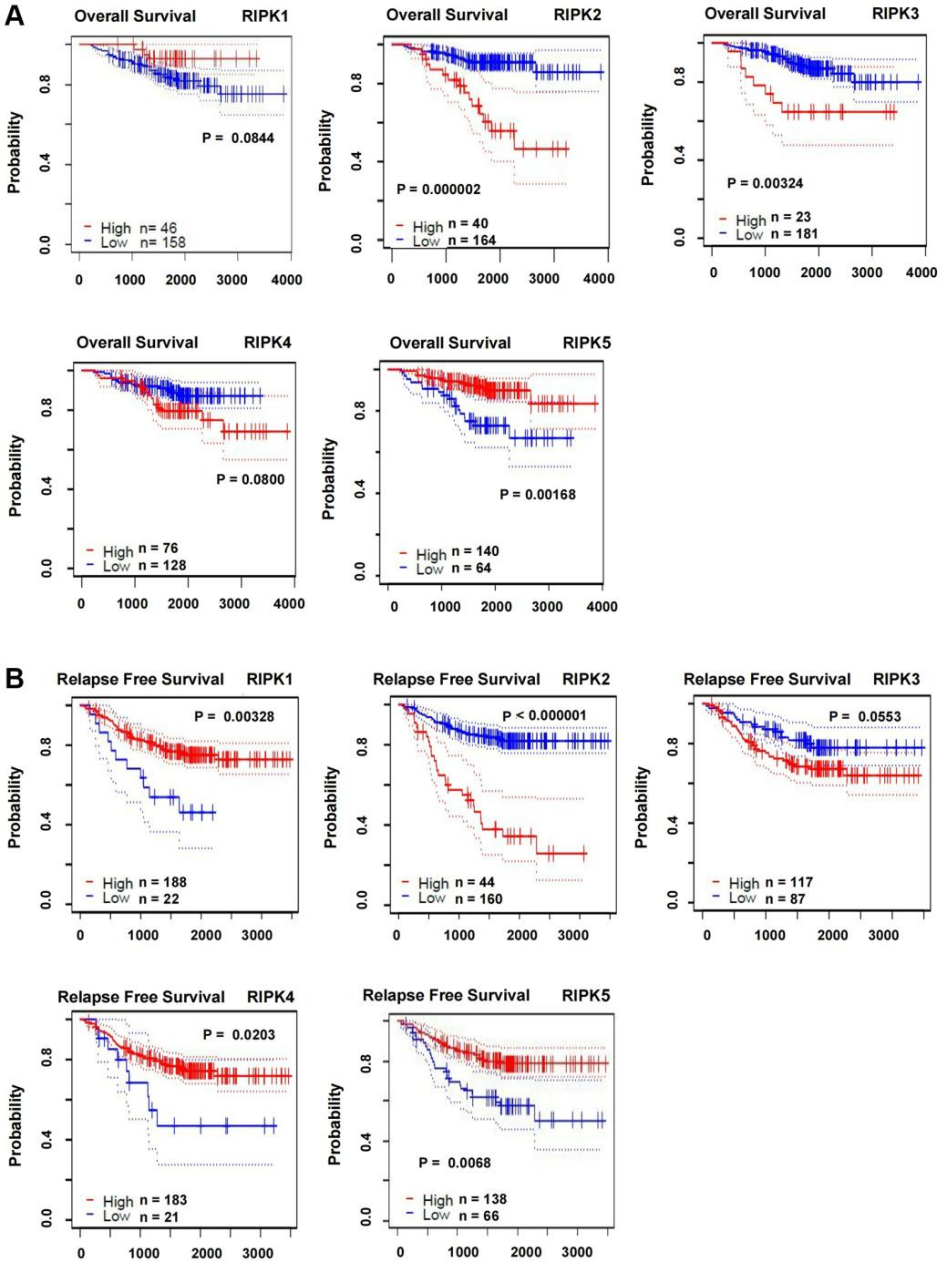
**The correlations of RIPK family expression with OS and RSF in LUAD patients.** (**A**) Kaplan-Meier plotter was used to assess the correlation of RIPK family members with the patients’ OS. (**B**) The correlations of RIPK family expression with RFS in LUAD patients.

Furthermore, we analyzed the impact of RIPK family on the survival of LUAD patients base on the PrognoScan databases. The overall survival (OS) was displayed in Figure 3A in which worse prognosis were found in cases with higher level of RIPK2 (*p* < 0.00001) and RIPK3 (*p* = 0.00324), but higher level of RIPK5 suggested a longer survival time (*p* = 0.00168). In addition, relapse free survival (RFS) data was also estimated according to the RIPK family level. As showed in Figure 3B, high level of RIPK2 indicated a worse RFS (*p* < 0.00001), as well as the low level of RIPK1 (*p* = 0.00328), RIPK4 (*p* = 0.0203) and RIPK5 (*p* = 0.0068).

### Genetic alteration and methylation level of the RIPK family in LUAD patients

**Figure 4:**
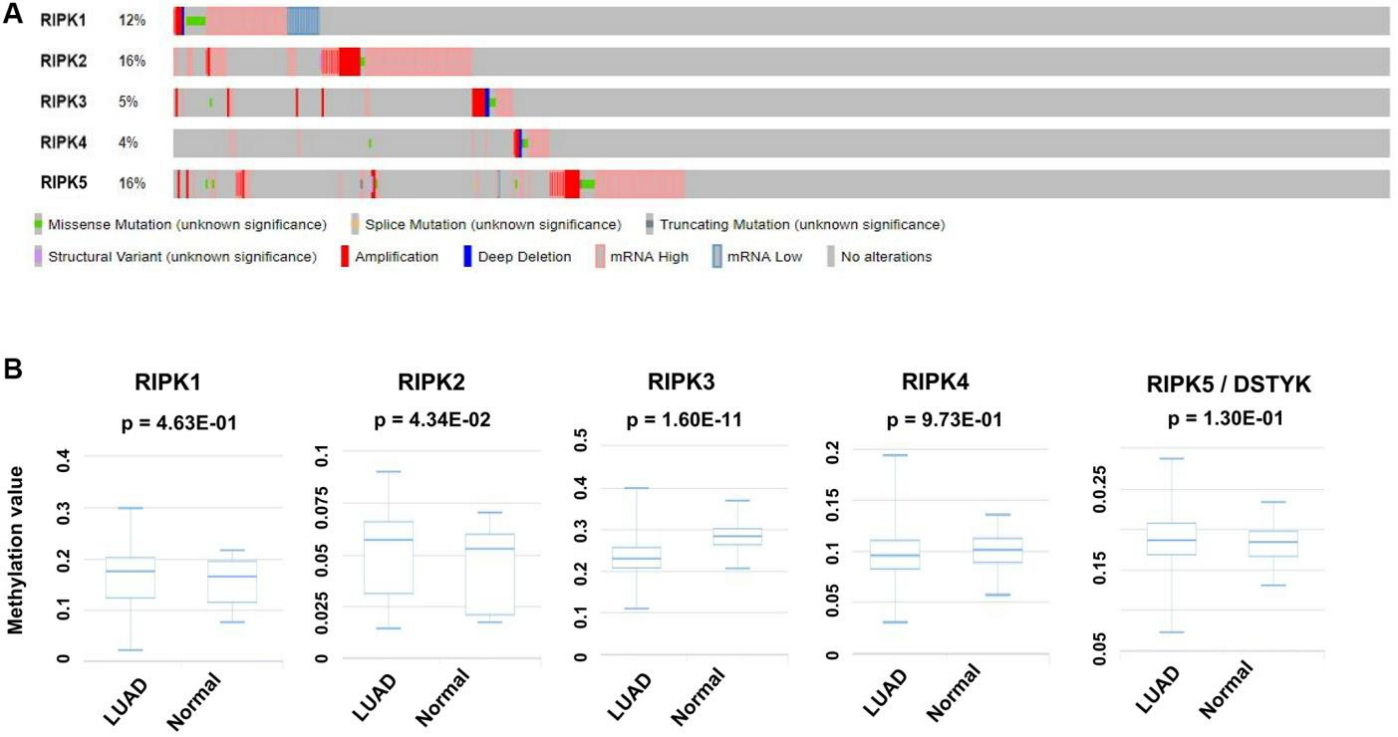
**Genetic alteration and methylation level of the RIPK family in LUAD patients.** (**A**) Genetic alteration of the RIPK family in LUAD patients analyzed with cBioPortal. (**B**) The methylation values of RIPK family members were evaluated using the DiseaseMeth database.

Genetic alteration is another pivotal factor resulting in tumor generation and advanced progression. We herein analyzed the alteration profiles of RIPK members by using the cBioPortal database. RIPK2 and RIPK5 present highest alternation ratio reaching at 16% among the LUAD cases, in which mutation was rich in ‘mRNA high’ annotation. The genetic mutations of RIPK1, RIPK3 and RIPK4 were 12%, 5% and 4% respectively (Figure 4A).

Methylation level was supposed as another regulation mechanism involving in LUAD. From DiseaseMeth database related to LUAD cases, we could confirm a higher methylation level of RIPK2 (*p* = 0.0434), but lower methylation level of RIPK3 (*p* = 1.60e-11) when comparing to health people (Figure 4B).

### Interaction and functional enrichment analysis of the RIPK family in LUAD patients

**Figure 5:**
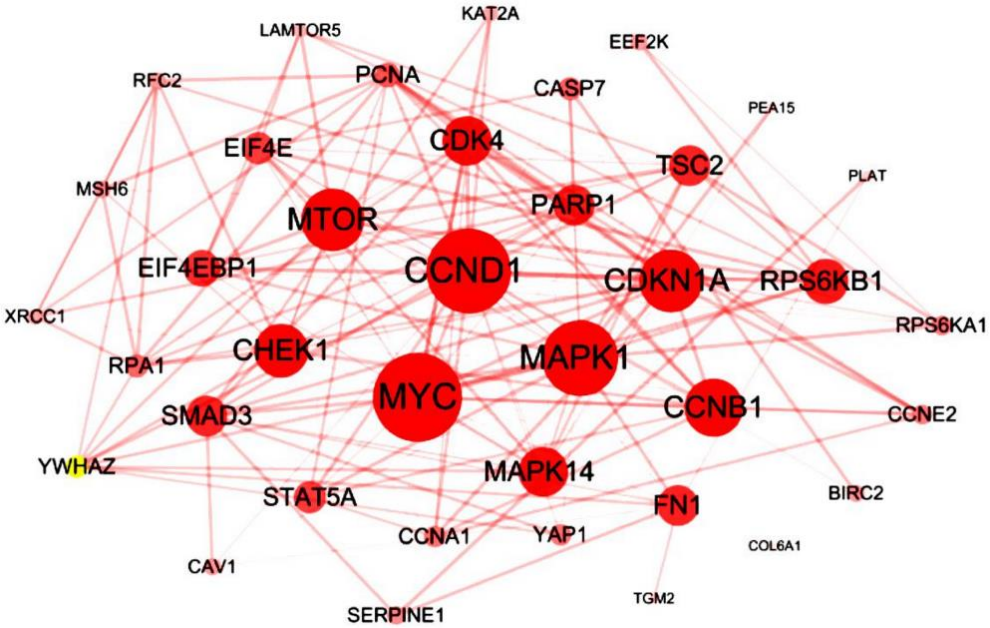
**Interaction analysis of the RIPK family in LUAD patients.** The 42 most frequently altered genes identified from cBioPortal that are linked to the RIPKs family in LUAD patients.

We analyzed cBioPortal database and finally extracted 42 most frequently altered genes which mRNA levels were significantly correlated to the RIPK members in LUAD patients. Several hub genes, including CCND1, MYC, MTOR, MAPK1, CDKN1A and CCNB1, were turned out to participate actively in the bio-malignant behaviors of RIPK family modulation in LUAD cases (Figure 5).

**Figure 6:**
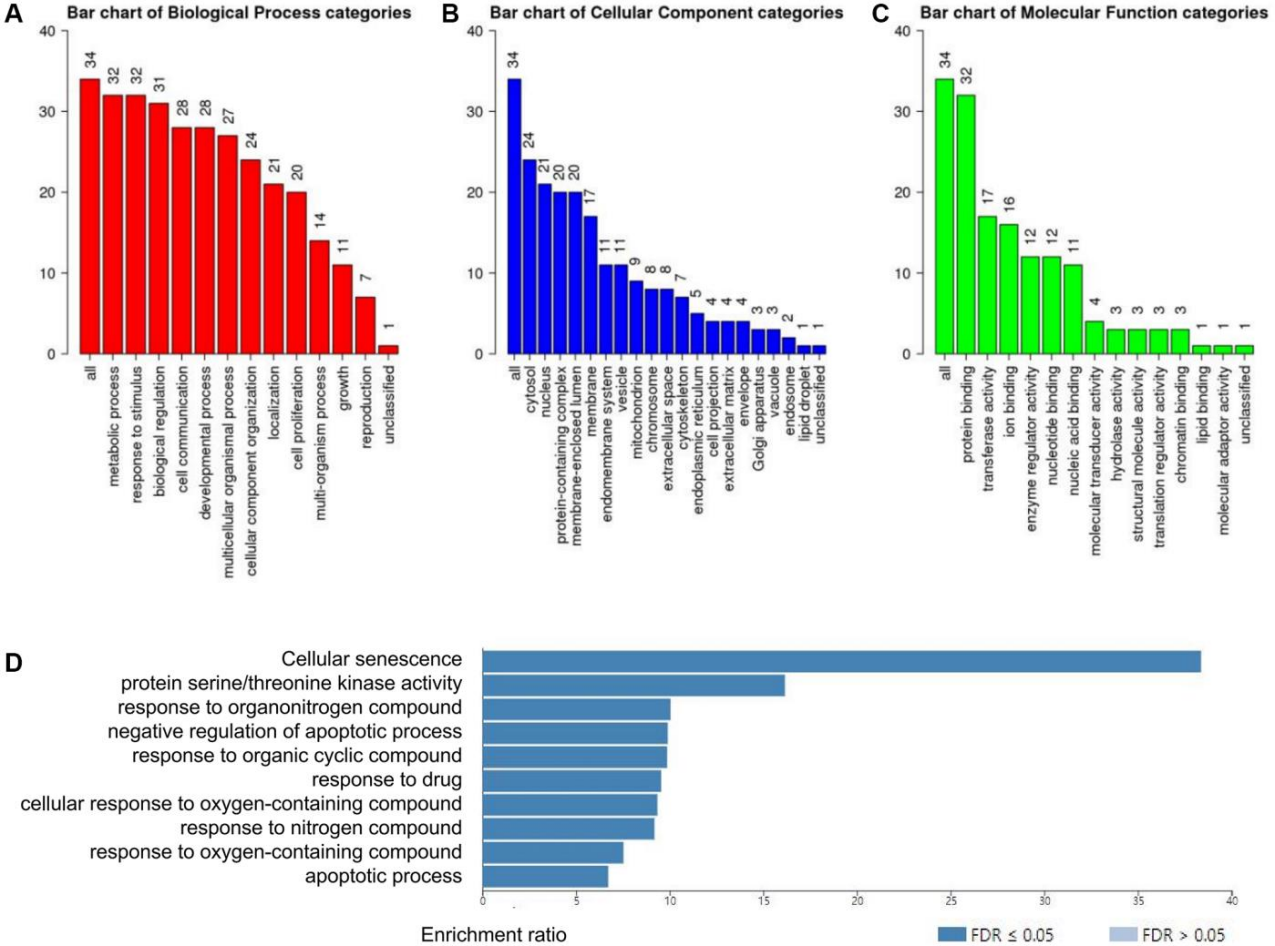
**Functional enrichment analysis of the RIPK family in LUAD patients with WebGestalt database.** (**A**–**C**) Bar plot of GO enrichment in cellular components, biological processes, and molecular functions. (**D**) The bar plot of KEGG enrichment.

Next, WebGestalt database was applied to estimate the biological functions of RIPK family based on the strong relevant genes above. As shown in the GO pathways, the top enriched biological processes were metabolic process, response to stimulus, biological regulation and cell communication (Figure 6A). Cellular component categories were highly enriched in cytosol, nucleus protein-containing complex, membrane-enclosed lumen, membrane (Figure 6B). When involving the molecular function categories, the top five related function were protein binding, transferase activity and ion binding (Figure 6C).

In addition, 10 KEGG pathways was strongly associated to the biological functions of RIPK family in the generation and progression of LUAD, including cellular senescence, protein serine/threonine kinase activity, response to organonitrogen compound, negative regulation of apoptotic process, response to organic cyclic compound, response to drug, cellular response to oxygen-containing compound, response to nitrogen compound, response to oxygen-containing compound and apoptotic process (Figure 6D).

### Immune cell infiltration of the RIPK family

**Figure 7:**
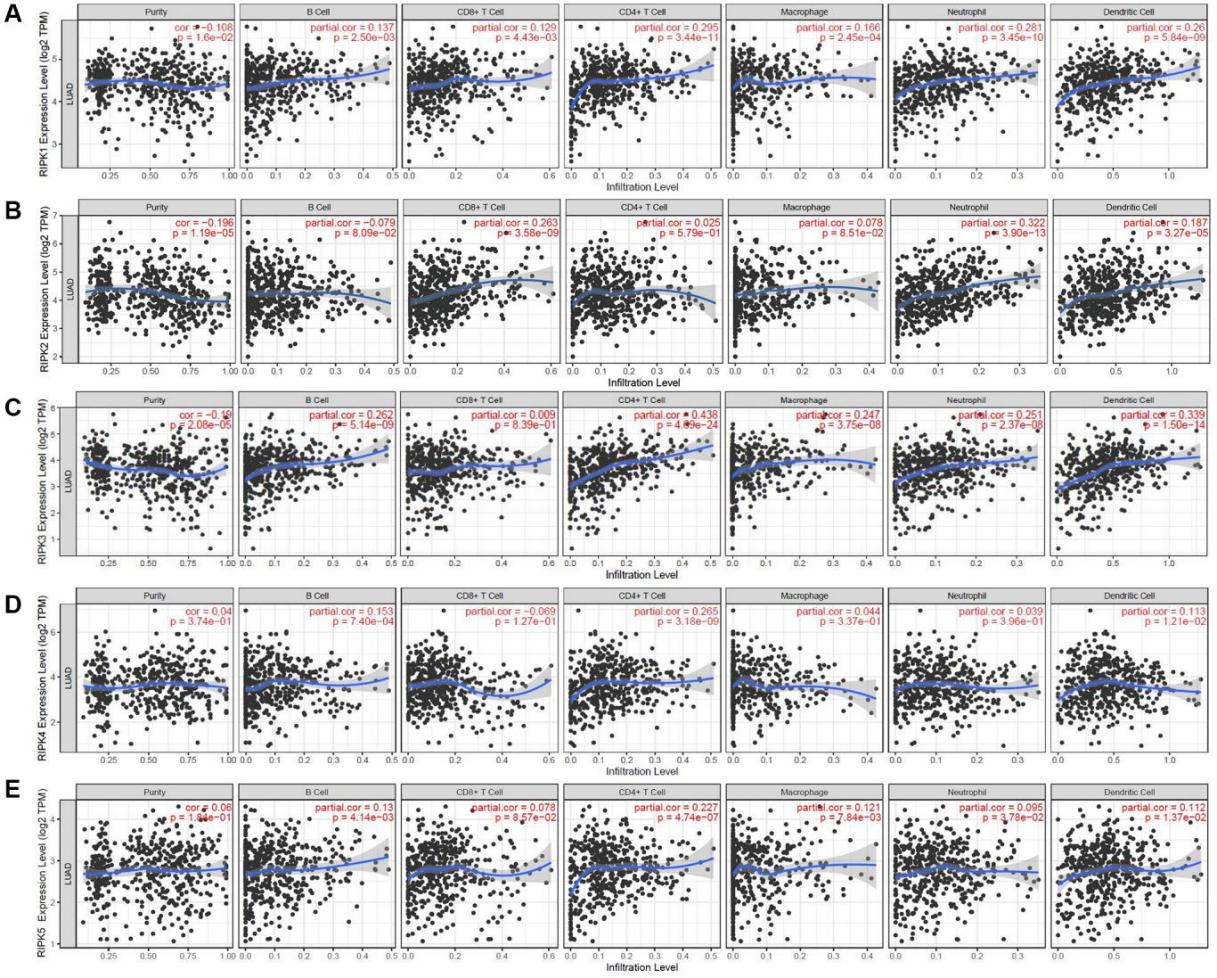
**The relationship between immune cell infiltration and the expression of the RIPK family.** The TIMER database was used to analyze the effect of (**A**) RIPK 1, (**B**) RIPK 2, (**C**) RIPK 3, (**D**) RIPK 4, (**E**) RIPK 5 on the abundance of immune cells in LUAD patients.

Recent study illustrated that the roles of immune cells should be seriously considered in the LAUD progression, as well as their impacts on treatment efficiencies and prognosis. Hence, we identified the connection between RIPK family members and six distinct immune subsets which infiltrated tumor via TIMER database analysis. Intriguingly, RIPK1 was positively linked to the infiltrated B cell (Cor = 0.137, *p* = 2.50e-03), CD8+ T cell (Cor = 0.129, *p* = 4.43e-03), CD4+ T cell (Cor = 0.295, p=3.44e-11), macrophage (Cor = 0.166, p=2.45e-04), neutrophil (Cor = 0.281, *p* = 3.45e-10), dendritic cell (Cor = 0.26, *p* = 5.84e-09) (Figure 7A). RIPK2 level was positively related to the infiltrated CD8+ T cell (Cor = 0.263, *p* = 3.58e-09), neutrophil (Cor = 0.322, *p* = 3.90e-13), dendritic cell (Cor = 0.187, *p* = 3.27e-05) (Figure 7B). Further, positive correlation was spotted between RIPK3 and B cell (Cor = 0.262, *p* = 5.14e-09), CD4+ T cell (Cor = 0.438, *p* = 4.09e-24), macrophage (Cor = 0.247, *p* = 3.75e-08), neutrophil (Cor = 0.251, *p* = 2.37e-08), dendritic cell (Cor = 0.339, *p* = 1.50e-14) (Figure 7C). For RIPK4, we found its mRNA level positively related to infiltrated B cell (Cor = 0.153, *p* = 7.40e-04), CD4+ T cell (Cor = 0.265, *p* = 3.189e-09), dendritic cell (Cor = 0.113, *p* = 1.21e-02) (Figure 7D). Finally, expression of RIPK5(DSTYK) showed its positive relationship to B cell (Cor = 0.13, *p* = 4.14e-03), CD4+ T cell (Cor = 0.227, *p* = 4.74e-07), macrophage (Cor = 0.121, *p* = 7.84e-03), neutrophil (Cor = 0.095, *p* = 3.78e-02), dendritic cell (Cor = 0.1126, *p* = 1.37e-02) (Figure 7E).

The relationship between RIPK family and the infiltration of immune cells were analyzed (Table 2). 12 confounding factors (macrophages, neutrophils, dendritic cells, B cells, CD8+ T cells, CD4+ T cells and five RIPK family members) were put into the Cox proportional hazard model together for further analyzed. From Table 2, we found that B cells (HR = 0.012, *p* = 0.001), CD4+ T cells (HR = 29.495, *p* = 0.0170) and RIPK2 (HR = 1.458, *p* = 0.001) could be considered as independent prognosis factors related to LAUD.

**Table 2:** The cox proportional hazard model of RIPK family and six types of immune cells in LUAD patients from TIMER database.

	**Coef**	**HR**	**95% CI**	***P* value**	**Sig.**
B cell	−4.432	0.012	0.001−0.161	0.001	^**^
CD8 T cell	−0.165	0.848	0.131−5.500	0.863	
CD4 T cell	3.384	29.498	1.842−472.264	0.017	^*^
Macrophage	0.664	1.943	0.143−26.363	0.618	
Neutrophil	−2.275	0.103	0.002−5.513	0.263	
Dendritic cell	−0.208	0.812	0.214−3.077	0.759	
RIPK1	−0.066	0.936	0.639−1.372	0.734	
RIPK2	0.377	1.458	1.168−1.821	0.001	^**^
RIPK3	−0.138	0.871	0.702−1.080	0.209	
RIPK4	−0.066	0.937	0.772−1.136	0.506	
DSTYK	−0.066	0.936	0.695−1.257	0.662	

## DISCUSSION

RIPKs share homologous kinase domains but differ in other functional units, such as identical P-loops, an ionic pair of catalytic Lys and Glu residues in the center of C helix, and an HXD motif in the catalytic loop [1, 4, 12]. Thus, the core kinase domain and these various elements endow these proteins with multiple functional connections and distinct clinical properties in cancer, as supported by the data presented in our study related to LUAD.

RIPK1 were proved as a core protein in a wide range of aberrant physiological processes like neurodegeneration, autoimmune, inflammatory activation and malignancies [13, 14]. Aberrantly expressed RIPK1 has been demonstrated to regulate the cell death pathways, including apoptosis [15] and necrotic cell death [16] as well as ferroptosis [5]. The underlying pathway always leads to activation of MAPKs and NF-κB [17], along with phosphorylation [18] and ubiquitination [19]. RIPK1 participates in various cancers and induces malignancy. Activation of the RIPK1/NF-κB pathway can induce NSCLC cell proliferation and migration [20]. Single-nucleotide polymorphism rs17548629 in RIPK1 may be associated with the formation of lung cancer [21]. However, whether this molecule can be considered a target for tumor therapy is still a matter of dispute. Patel et al. did not find any effect of a RIPK1 inhibitor on tumor growth or metastases, although it did help in inflammatory disease control [22]. Our data revealed that RIPK1 expression was decreased in LUAD and that it increased RFS.

RIPK2 is also known as RIP-like-interacting caspase-like apoptosis-regulatory protein kinase or CARD-containing IL-1b converting enzyme-associated kinase [1, 23]. RIPK2, as a critical protein for the signaling from NOD-like receptors, always triggered MAPK, ERK2, and JNK activation and further affect apoptosis [24]. Thus, target treatment on RIPK2 showed an ideal control of inflammatory diseases, such as cystic fibrosis, asthma, inflammatory bowel disease and pancreatitis [3, 25, 26]. Rare study has documented RIPK2 functions and roles in LUAD. According to our analysis, higher RIPK2 expression was observed in LUAD tissues, suggesting a worse prognosis, and this was positively correlated with the pathological stage. Notably, higher methylation levels of RIPK2 were also found in tumor tissues. Generally, DNA methylation indicates resilience of gene transcription [27]. Here, both RIPK2 expression and methylation levels increased in tumor tissues, which may reflect DNA methylation, not only in the intergenic region or CpG islands to repress gene transcription but also in the non-first exon of the gene body, which is associated with a higher level of gene expression in dividing cells [27]. Surprisingly, the Cox proportional hazard model identified RIPK2 as an independent prognostic factor, as well as B cells and CD4+ T cells. The TIMER database showed that RIPK2 is positively linked to infiltration by B cells, CD8+ T cells, neutrophils, and dendritic cells. This suggests that RIPK2 is a potential target and immunoregulatory protein in LUAD patients.

Similar to RIPK1, RIPK3 play a key function for caspase-independent necrotic cell death via anti-TNF treatment [17, 28]. Phosphorylation of RIPK1 and RIPK3 can stabilize their interaction within the pronecrotic complex inducing kinase activity, triggering downstream ROS (reactive oxygen species) reaction, and resulting in terminal necrotic cell death [29]. However, there are still some divergent opinions regarding its function. Newton et al. reported that the level of NF-kB activation could be induced by TNF in RIPK3-deficient mice [30]. RIPK3 is thought to be a carcinogenic factor in various cancers, such as breast cancer, intestinal and colon cancer, and lung cancer [31]. According to our data, RIPK3 expression negatively correlated with prognosis and positively correlated with various tumor-infiltrating lymphocytes. We also found a lower methylation level of RIPK3 in LUAD patients, which suggests that epigenetic regulation participates in RIPK3-induced biological properties of LUAD.

In addition to sharing a kinase domain, RIPK4 has a C-terminal ankyrin repeats and N-terminal kinase domain part [4]. RIPK4 strongly links to the NF-κB and JNK signaling activation [32] and also regulates the PKC signaling [4]. A recent study revealed that RIPK4 can influence the biological properties of malignancies, such as pancreatic [33], bladder [34], and cervical cancers [35]. It can inhibit STAT3 signaling to sustain LUAD differentiation [36]. Our data revealed that RIPK4 levels are increased in LUAD samples, based on GEPIA and UCLCAN. In addition, RIPK4 was found to positively correlated with infiltrating CD4+ T cells, which has not been reported by other groups.

RIPK5 (encoded by DSTYK) exhibits high sequence homology with RIPK4 (with an overall level of homology of 35%), suggesting similar functions [1]. Increase of RIPK5 could induce cell apoptosis via DNA fragmentation [37]. However, the biological function of RIPK5 remains largely unknown. Our research showed that RIPK5 expression was low in LUAD patients and was indicative of a better prognosis. Additionally, the level of infiltrating B cells and CD4+ T cells were both strongly related to the expression of RIPK5.

In conclusion, we presented the expression profiles of RIPK family members in patients with LUAD. Those findings may facilitated identification of new diagnostic markers and treatment targets, thereby leading to new treatment options for LUAD patients to prevent tumor relapse and prolong their survival time.

## MATERIALS AND METHODS

### GEPIA/Wanderer/UALCAN database

With a web server calculation, Gene Expression Profiling Interactive Analysis (GEPIA) database can illustrate us the gene expression difference and interactive relationship between cancer and normal tissue [38]. The Wanderer database can visualize gene expressions according to TCGA data [39]. UALCAN could offer researchers a group of comprehensive and interactive web data via analyzing cancer bio-information data [40]. We used these databases to estimate the expression level of RIPKs genes and also the roles of the RIPK family in clinic stage, prognosis as previous description [41]. *P*-values was set at 0.05 in our process.

### PrognoScan database

We use PrognoScan database [42] to analyze and illustrate the expression RIPK family members and the survival trend in LUAD patients. Here, overall survival (OS) and relapse-free survival (RFS) curves were included to evaluate the prognosis of patients with LUAD. *P*-value will offer if less than 0.05.

### cBioPortal

cBioPortal database includes more than 100 malignancies genomics data. With a series user-friendly calculation strategy, researchers can obtain the genetic alterations and the coexpression profiles conveniently [43]. In this study, we focus on the data related to the RIPK family in LUAD tissue.

### STRING

STRING was a kind of website which was always applied to dig out potential protein-protein interactions (PPIs) [44]. In this study, RIPK family member-associated protein-protein interactions relationship data in detail were download and then a PPIs network were constructed with Cytoscape software [45].

### DiseaseMeth

DiseaseMeth is one of the database which can visualize the DNA methylation level with gene expression [46]. In our analysis, the relationship between the expression of the RIPK family and their methylation levels were sought out.

### WebGestalt

The aims of WebGestalt is providing a better understanding of functions of gene set [47], like Gene Ontology (GO) and Kyoto Encyclopedia of Genes and Genomes (KEGG) rerichment. Here, we analyzed the enrichment pathways associated with the RIPK family in the patients with LUAD.

### TIMER

TIMER is a useful database to evaluate the immune cells infiltration status among various cancers, offering an immune strategies and targeting molecules clues [48]. Infiltrated immune cells features and RIPKs expression were drew out via the TIMER database.
